# Applicability of a nationwide flood forecasting system for Typhoon Hagibis 2019

**DOI:** 10.1038/s41598-021-89522-8

**Published:** 2021-05-13

**Authors:** Wenchao Ma, Yuta Ishitsuka, Akira Takeshima, Kenshi Hibino, Dai Yamazaki, Kosuke Yamamoto, Misako Kachi, Riko Oki, Taikan Oki, Kei Yoshimura

**Affiliations:** 1 IIS, The University of Tokyo, Tokyo, Japan; 2 University of Massachusetts, Amherst, USA; 3 Japan Aerospace Exploration Agency (JAXA), Tsukuba, Japan; 4 United Nations University, Tokyo, Japan; 5 Graduate School of Engineering, The University of Tokyo, Tokyo, Japan

**Keywords:** Environmental sciences, Environmental social sciences, Hydrology, Natural hazards

## Abstract

Floods can be devastating in densely populated regions along rivers, so attaining a longer forecast lead time with high accuracy is essential for protecting people and property. Although many techniques are used to forecast floods, sufficient validation of the use of a forecast system for operational alert purposes is lacking. In this study, we validated the flooding locations and times of dike breaking that had occurred during Typhoon Hagibis, which caused severe flooding in Japan in 2019. To achieve the goal of the study, we combined a hydrodynamic model with statistical analysis under forcing by a 39-h prediction of the Japan Meteorological Agency's Meso-scale model Grid Point Value (MSM-GPV) and obtained dike-break times for all flooded locations for validation. The results showed that this method was accurate in predicting floods at 130 locations, approximately 91.6% of the total of 142 flooded locations, with a lead time of approximately 32.75 h. In terms of precision, these successfully predicted locations accounted for 24.0% of the total of 542 locations under a flood warning, and on average, the predicted flood time was approximately 8.53 h earlier than a given dike-break time. More warnings were issued for major rivers with severe flooding, indicating that the system is sensitive to extreme flood events and can issue warnings for rivers subject to high risk of flooding.

## Introduction

As one of the most frequently occurring natural disasters, floods threaten millions of people and significantly damage socioeconomic development. Under the current warmer climate, flood risks have increased in most of the world^1–3^. Japan has lost billions of homes and businesses and hundreds of lives to frequent typhoons. According to the Japan Meteorological Agency (JMA), approximately 799 typhoons approached Japan, with 206 landing in the country from 1951 to 2019. In 2019, Typhoon Hagibis swept central, eastern, and northern Japan from October 11 to 13. This typhoon increased the damage caused by Typhoon Faxai, which destroyed most of the residential regions in Chiba Prefecture (eastern part of Japan). The typhoon’s trajectory covered 15 prefectures, and heavy rain warnings were issued in these regions. It resulted in 86 deaths, three missing persons, nearly 500 people injured, and approximately 400 billion dollars of damage. According to reports from the Ministry of Land, Infrastructure, Transport and Tourism (MLIT), 142 locations sustained structural damage, such as dike failure (https://www.mlit.go.jp/common/001313204.pdf). This enormous disaster was explained by a sufficient supplement of precipitable water under very humid conditions^4^, which caused a strong convergence of runoff in comparison with other extreme flood events, such as the 2018 event^5^.

In Japan, most rivers and river reaches are closely associated with densely populated regions. These regions become vulnerable when flooding occurs due to heavy rainfall associated with events such as typhoons and storms. Floods are the inevitable rapid and dangerous results of typhoon events because most urban areas lie on a floodplain. However, no effective river flood forecasting method is available to provide a sufficiently long warning time with high accuracy. The MLIT provides accurate predictions with a few hours of warning before flooding occurs based on the observed upstream water level, but this is too late for people to respond effectively, and the situation is even worse if flooding occurs late in the night. Therefore, precise forecasting with longer lead times is extremely important for densely populated, low-elevation coastal regions. Furthermore, as Japan is mountainous and includes many small basins, a validation study is needed to determine whether numerical flood forecasting can be effective in such a challenging region. However, no numerical flood forecasting system has been tested in Japan.

Several flood forecasting systems, with different lengths of lead time, have been developed to cover global or regional scales^6,7^. For example, at the global scale, there are two flood forecasting systems: the Global Flood Forecasting and Information System (GLOFFIS) run by Deltares^8^ and the Global Flood Awareness System (GloFAS)^9^ developed jointly by the European Commission and the European Centre for Medium-range Weather Forecasts. In addition to GLOFFIS and GloFAS, the Global Flood Monitoring System (GFMS) aims to produce real-time global maps of flood events^10,11^. There are also several regional-scale forecasting systems, such as the European Flood Awareness System (EFAS) of the European Commission^12^, the Hydrologic Ensemble Forecasting Service (HEFS) covering the continental USA by the U.S. National Weather Service^13^, Hydrological Predictions for the Environment (E-HYPE) operated by the Swedish Meteorological and Hydrological Institute^14^, and the Flood Forecasting and Warning Service (FFWS) run by the Bureau of Meteorology of Australia. Among these flood forecasting systems, GloFAS can achieve prediction lengths in excess of 25 days for some large basins and up to 20 days for some small basins^15^. In comparison with global systems, HEFS, covering the continental USA, strives to forecast with a longer prediction length of up to 1 week^6^, EFAS issues a national warning with lead times of up to 2 days, and the Bureau of Meteorology of Australia issues warnings with a minimum prediction length of 6 h. The wide range of forecasted prediction lengths given by these systems depends on the target of the systems. If the target is decision support for evacuation, high accuracy is required, so the prediction length can be short. However, if the target is early warning to improve preparedness among citizens, longer prediction length is prioritized over accuracy.

More specifically, in the case of flood forecasting in Japan, as stated above, alarms issued by the MLIT have reasonably high accuracy but short prediction length because their goal is to obtain precise and specific flood control locations to evacuate citizens. It is important that citizens are prepared for disaster, so a system with longer prediction length but relatively low prediction accuracy might be desirable, but Japan has no other official flood forecasts due to legal restrictions.

Furthermore, the prediction lengths of the aforementioned systems are clearly related to the predictability of the target, i.e., the lead time, so the definition of lead time is dependent on the catchment structure and the forecasting and warning system facilities^16^. Therefore, there is no specific way to validate predictability, which hinders improvements to flood forecasting systems through assessments of the accuracy of forecasting results^17^. As an indicator of flooding, the time of a dike break can directly distinguish between a flooded and non-flooded area and thus provide valid information about the time of flooding.

**Figure 1:**
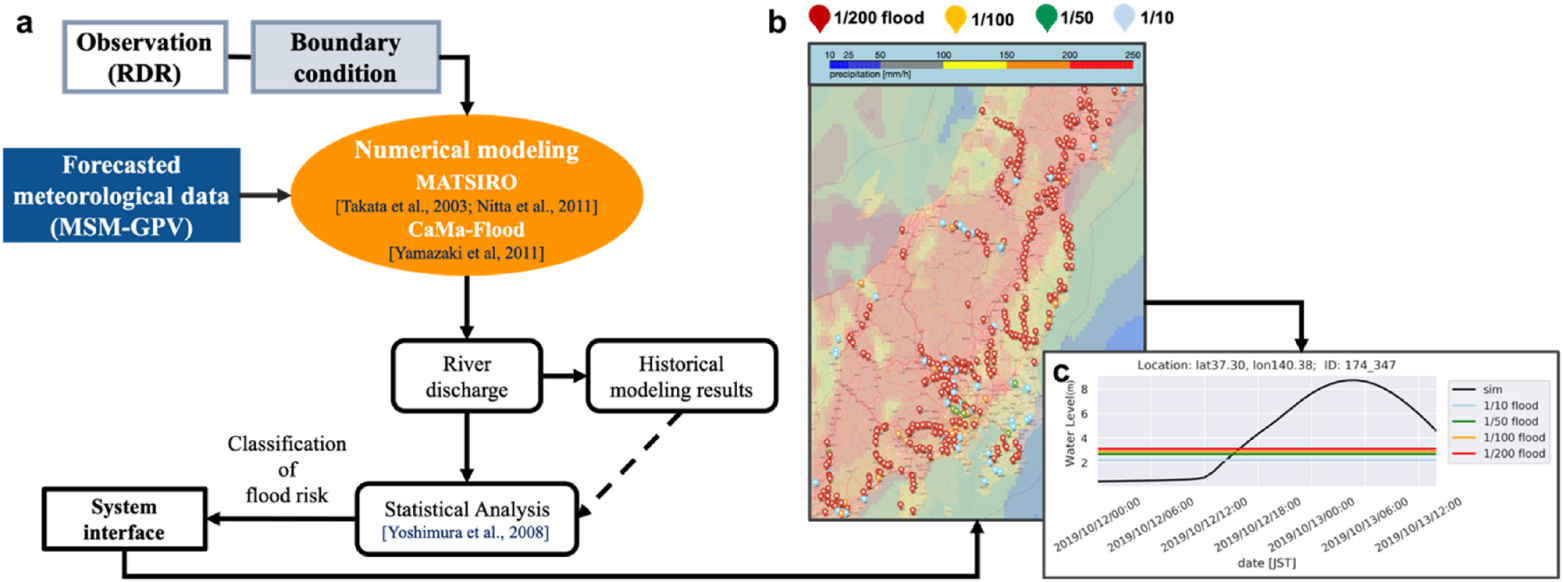
Schematic of the flood forecasting system. (**a**) Flowchart of the flood forecasting system. (**b**) Snapshot of the system interface at 00:00 JST on Oct. 12, 2019. Pins of different colors represent once per 200-year floods (red) and once per 100-, 50-, and 10-year floods (orange, green, and light blue, respectively). The real-time forecasting interface can be accessed at http://apps.diasjp.net/tdjpn/training/latest/jpn.html. (**c**) The forecasted 1/200-year hydrograph starting from 00:00 JST on Oct. 12, 2019 at 140.38° N, 37.30° E. This figure was generated through Python2.7 (https://www.python.org/), and Microsoft PowerPoint Version 16.47 provided by University of Tokyo.

Here, we newly present a flood forecasting system with longer prediction length developed by Ishitsuka^18,19^ and Yoshimura et al.^20^. Flood forecasting results of Typhoon Hagibis in 2019 were validated using the forecast flooding time and all dike-break times for flooded locations. Specifically, we used 39-h predictions of the JMA Meso-Scale Model grid point values (MSM-GPVs) as forcing data to run a land surface model, the Minimal Advanced Treatments of Surface Interaction and RunOff (MATSIRO) model, to obtain runoff values (Fig. 1a). MATSIRO is a physical-based land surface model, and the simulation covers a horizontal resolution of approximately 5 km (0.05 degrees) from 24° to 46° N latitude and 123° to 148° E longitude in Japan. Then, we employed a catchment-based macroscale floodplain model (CaMa-Flood) to estimate river water depth and flood area for all rivers and streams with an approximately 5-km (0.05 degrees) horizontal resolution. CaMa-Flood calculates the river discharge of a 1-dimensional river channel. The river parameters were calculated from Multi-Error-Removed Improved-Terrain (MERIT) DEM and hydrography (MERIT Hydro)^21,22^ datasets. Subsequently, the statistical distribution of river water depth given by CaMa-Flood was analyzed for comparison with return period values used for generating flood alarms. Here, the Gumbel distribution^23–27^ was applied because of its better fitting for extreme value analysis, such as values of extreme flood events^26,28^.

## Results

### Simulated flood locations and flood time

Figure 1 shows the procedures of this forecasting system and a screenshot of the interface featuring Typhoon Hagibis. The red pins are flood alarms that occurred at 00:00 JST on October 12 (Fig. 1b). These alarms are updated every 3 h. For each alarm, a hydrograph is archived to show the exact flooding alarms for 1/10-, 1/50-, 1/100-, and 1/200-year return periods (Fig. 1c). To obtain the forecasting results, we first applied numerical modeling to a 39-h forecasting dataset comprising MSM-GPVs to force the land surface model MATSIRO^29^ and the hydrological model CaMa-Flood^30^. Then, the estimated river water depth was analyzed via comparison with the return period. In this study, we chose locations with a return period of 200 years as forecast locations because the occurrence of flood levels in major Japanese rivers is typically set to once during a 100–200-year event (Fig. 1b,c). This method was first tested by Yoshimura et al.^20^, who assessed six river predictions in 2003 and 2004 using the previous version of the MSM-GPV dataset, in which 18-h predictions were made every 6 h.

### Dike-break time

To evaluate the forecasting performance, we obtained the dike-break times (DBTs) for all flood locations. We used dike breaks to represent all locations where flooding might occur with various inundation patterns related to the time of levee or river dike breakage. To obtain DBTs, we collected official reports, issued by the MLIT (https://www.ktr.mlit.go.jp/kisha/index00000134.html), of JMA disaster prevention information in XML format (http://agora.ex.nii.ac.jp/cps/weather/river/), as well as information from Twitter and personal websites. According to the public broadcaster Nippon Hoso Kyokai (NHK) and the MLIT, there were floods at 142 locations. Among these flood locations, only 80 records of DBTs could be found. This finding indicates that proper records were lacking for many of the floods or inundations that were identified.

**Figure 2:**
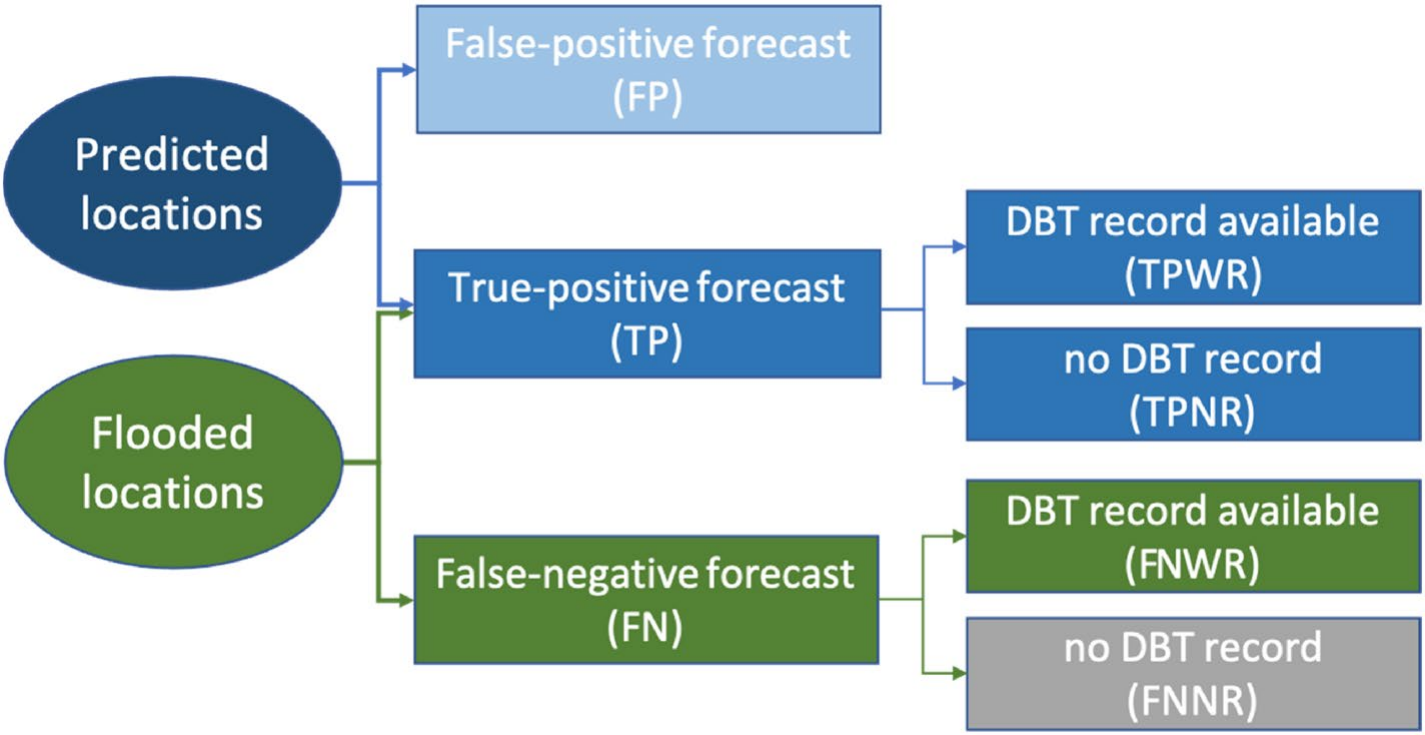
Classification of flood locations related to Typhoon Hagibis. This figure was generated through Microsoft PowerPoint Version 16.47 provided by University of Tokyo.

The classification scheme for predicted and flooded locations of Typhoon Hagibis are shown in Fig. 2. The predicted locations are locations with a more than 1/200-year-flood alarm issued by the flood forecasting system, and the flooded locations are areas where floods actually occurred. With reference to the DBT records, true positives (TPs), correctly forecasted locations, were further classified as true positives with DBT records (TPWRs) and true positives with no DBT records (TPNRs). Among the unsuccessfully predicted flood locations, only those with DBT records (FNWRs) were verifiable. Those locations determined to be incorrectly predicted with no DBT record (FNNRs) could not be verified by location, but they were traceable via flood reports.

**Figure 3:**
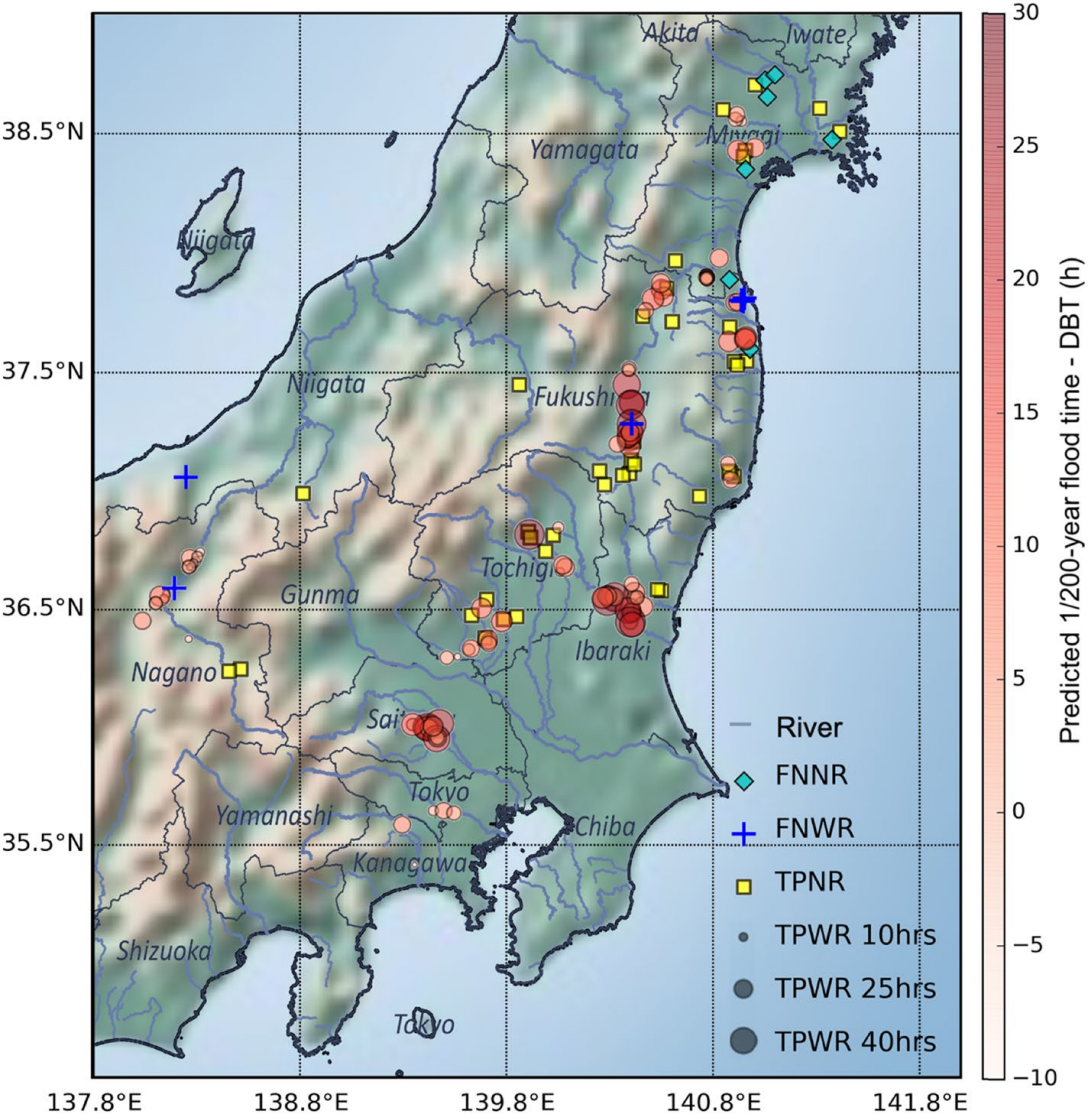
Forecasted locations and lead time distribution. FNNR indicates false-negative sites with no DBT records (blue diamonds, 8 spots). FNWR indicates false-negative locations with DBT records (blue crosses, 4 spots). TPWR (80 spots) and TPNR (50 spots) indicate locations that were successfully predicted (true positive) with and without DBT records, respectively. The color of the circle indicates how much the predicted 1/200-year flood time preceded the DBT at a given location. The size of the circle indicates the lead time in comparison with the predicted 1/200-year flood time. This figure was generated through Python2.7 (https://www.python.org/).

Figure 3 compares the forecasted 1/200-year flooding times and DBTs. We considered the following three outcomes: TPWR, FNNR, and FNWR. To compare the time differences between each predicted 1/200-year flooding time and DBT, we plotted these as color-gradient circles (Fig. 3). The circle size indicates the lead time for a given location, and the redness of the circles indicates the difference between the predicted 1/200-year flood time and the DBT. In the figure, locations with longer lead times are generally concentrated in the upper and middle reaches of rivers, where most flooding originate. We performed additional temporal analysis for the TPWR sites and found that the average lead time for the predicted 1/200-year flood time was approximately 32.75 h. Moreover, the predicted 1/200-year flood time was on average approximately 8.53 h earlier than the DBT, indicating that the predicted flood time was earlier than the real flood time. Because the goal of this system is to generate longer lead times, we argue that it is reasonable to accept this advancement, which is helpful because it allows more time for further evaluation and decision-making, such as for evacuation and disaster preparation.

Moreover, 12 locations (false negatives) were not predicted, but the occurrence of floods was recorded. Among these, five locations had DBT records (FNWR, blue crosses in Fig. 3), and seven sites had no DBT records (FNNR, green diamonds in Fig. 3), which were mainly located in Miyagi Prefecture and near downstream portions. It is reasonable to assume that the five FNWR sites were technical prediction failures, which means that 2.30% of the 142 flooded locations were not successfully predicted by this system. In addition to biases or uncertainties that might have been present in the forecasted meteorological data, the spatial resolution of this system, which was designed as 0.05 degrees and is thus relatively coarse for regional forecasts, was a potential source of these mispredictions. However, taking these false-negative locations (FNWRs and FNNRs) into account, this system successfully predicted 130 flood locations, approximately 91.55% of the 142 flood locations, with a gain of approximately 32.75 h of lead time.

### Merits of applying the forecasting system

**Figure 4:**
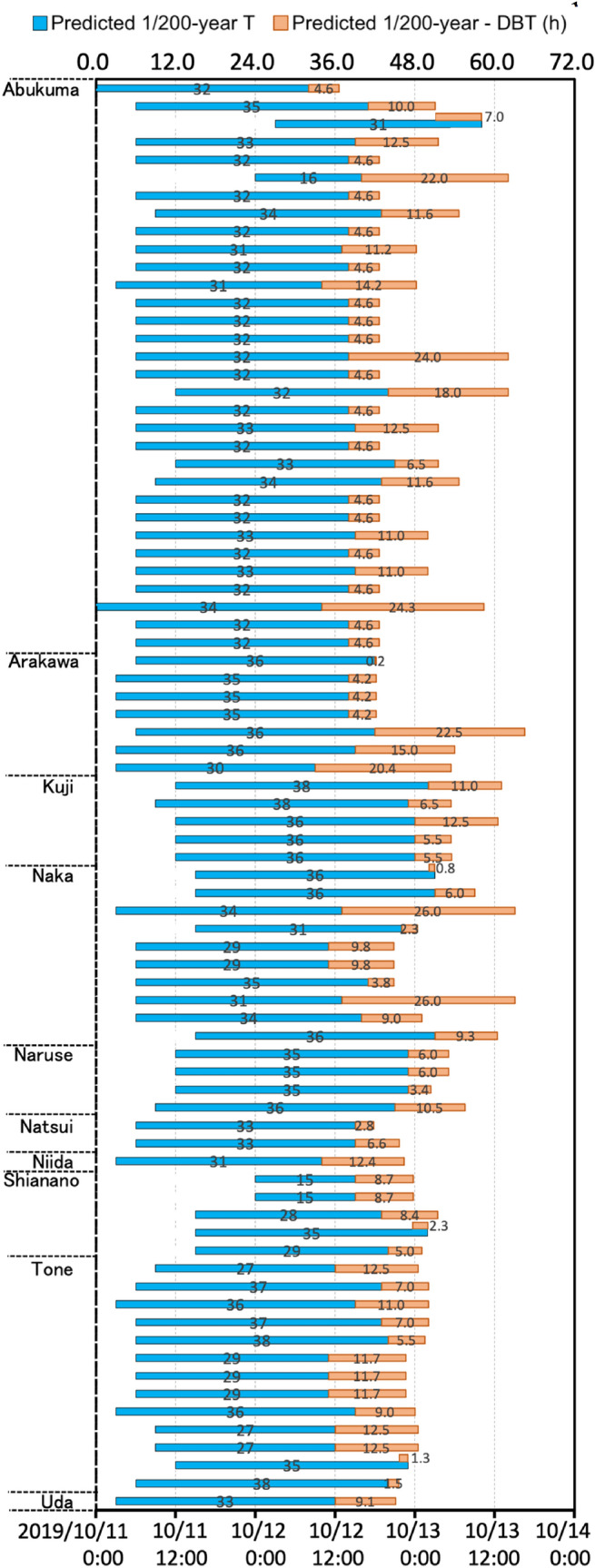
Comparison of predicted 1/200-year flood times and DBTs. The vertical axis shows the location of each flooded river. Each blue bar begins at the time when a 1/200-year flood was first predicted by the system. The end of each blue bar is the predicted flood time. The length of each blue bar is the lead time, which is approximately 32.75 h on average. The orange bars show differences between the DBT and the predicted 1/200-year flood time. The average of these differences indicates that the predicted 1/200-year flood time was approximately 8.53 h earlier than the DBT.

**Figure 5:**
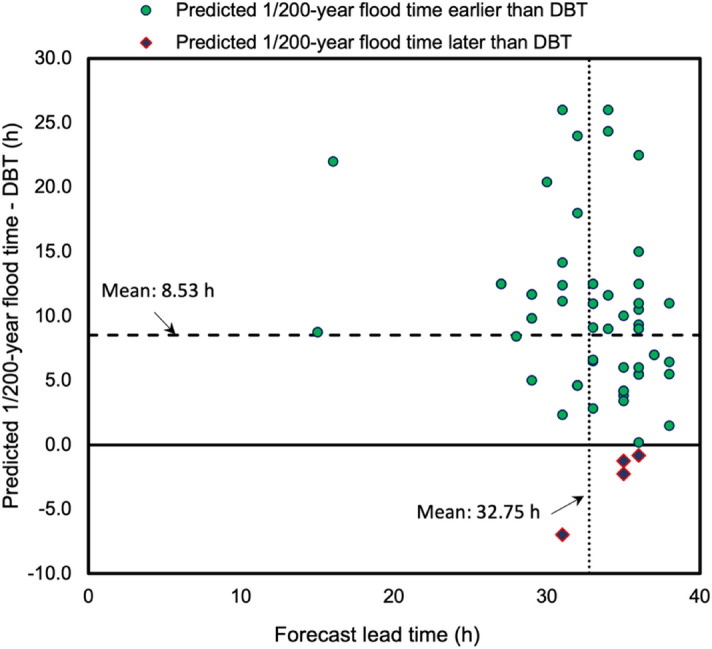
Differences between predicted 1/200-year flood times and DBTs, and the corresponding lead times. The vertical dashed line indicates the average lead time, which is approximately 32.75 h. The horizontal dashed line indicates the average difference between the 1/200-year flood time and the DBT, which is approximately 8.53 h.

This system has several merits. It can predict floods over an extensive number of locations, because some locations are not observed for various reasons, such as a lack of instruments, gaps in temporal observations, or dangers associated with collecting information. Moreover, because of flood damage, multiple problems may lead to a loss of observations. Therefore, one of the merits of a flood forecasting system is the provisioning of forecasting results without spatial or temporal limitations and the avoidance of physical risk. In Fig. 4, we show 80 locations (TPWR) from all predicted results for Typhoon Hagibis. Fifty TPNR locations did not have a DBT record (TPNR, Fig. 4). For example, in the Abukuma River, one of the hardest-hit areas^31^, 12 TPNR sites had no DBT records. As a result of this flood forecasting system, a warning time of more than 31.0 h could be achieved at these 12 TPNR locations, despite the fact that the physical detection method would not fully cover these areas. In the case of the Uda River (Fig. 4), flood alarms were issued at 3:00 a.m. on October 11, 2019. At that moment, the system predicted that 1/200-year flooding would occur at approximately 33.0 h later (at approximately 12:00 p.m. on October 12), which was validated as being approximately 9.1 h earlier than the DBT. Therefore, the actual lead time for the Uda River was approximately 42.1 h. However, warnings for 1/200-year floods forecasted at eight TPWR locations were issued later than the DBT by 2.3 h on average (Fig. 4). With the forecasted 33.0-h lead time for a 1/200-year flood, there was approximately 30.7 h of actual lead time for these eight TPWR locations. Figure 5 shows a comparison between 1/200-year flood times and DBTs, along with the lead times. Points representing a 1/200-year flood time later than the DBT are shown as diamonds with a red outline. On average, the flood times provided approximately 8.53 h of advanced warning relative to the DBT, with a predicted 32.75-h lead time. Overall, for 80 TPWR locations, this system provided at least 32.75 h of lead time. For some locations, such as flooded locations along the Abukuma River, Ara River, and Naka River, the actual lead time was more than 50 h. Furthermore, although we could not calculate the lead time for 50 TPNR locations due to the lack of observed DBTs, it is important to state that these floods were forecasted successfully by our system.

**Figure 6:**
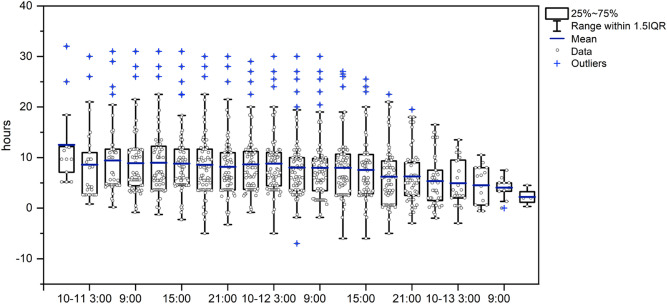
Difference between the predicted 1/200-year flood time and the DBT for each forecast for 80 TPWR locations. Quartile values and the mean across all TPWR locations are plotted for each estimation step.

In this flood forecasting system, estimations are conducted every 3 h. Therefore, we inspected the forecast lead time for each estimation between October 11 and 13, 2019 (Fig. 6). Differences between the predicted 1/200-year flood times and DBTs for each estimation period were plotted for all 80 TPWR locations. The earliest time when an alarm was issued was 00:00 on October 11, 2019. Differences between the predicted 1/200-year flood time and the DBT varied from − 7.2 h to approximately 32.0 h, with mean values varying from 3.7 to 11.6 h. As time passed, the mean values decreased, and the range of difference between the predicted 1/200-year flood time and the DBT decreased. This change indicates that the forecast accuracy is higher when the forecast time is closer to the time of typhoon landing.

## Discussion

This study performed temporal and spatial assessments of flood forecasting, which has been challenging to validate. Observing disasters remains a great challenge because the unpredictable and devastating effects may lead to missing in situ observations. Thus, flood forecast modeling is of particular importance. It is also capable of quantitatively estimating the water volume in each layer over the land surface, which is an advantage in comparison with satellite observations. Although the number of possible observations was limited, this system successfully predicted 91.55% (130/142) of the flood locations during Typhoon Hagibis, including 50 sites that had no recorded DBTs, with a lead time of approximately 32.75 h. We argue that this forecast lead time for flood locations is much longer than the lead time of traditional forecasts issued in Japan. The high accuracy demonstrated in this study will be critical for disaster preparation and evacuation. The forecast results also demonstrated that the combination of MSM-GPV forecasted forcing data, the MATSIRO land surface model, the CaMa-Flood hydrological model, and statistical analysis is an effective solution for predicting floods in Japan. It is also reasonable to expect that this method can be applied to flood forecasting in other regions with available forcing data. Furthermore, using ensemble forecasting may help improve reliability and identify uncertainties in forecasting.

**Figure 7:**
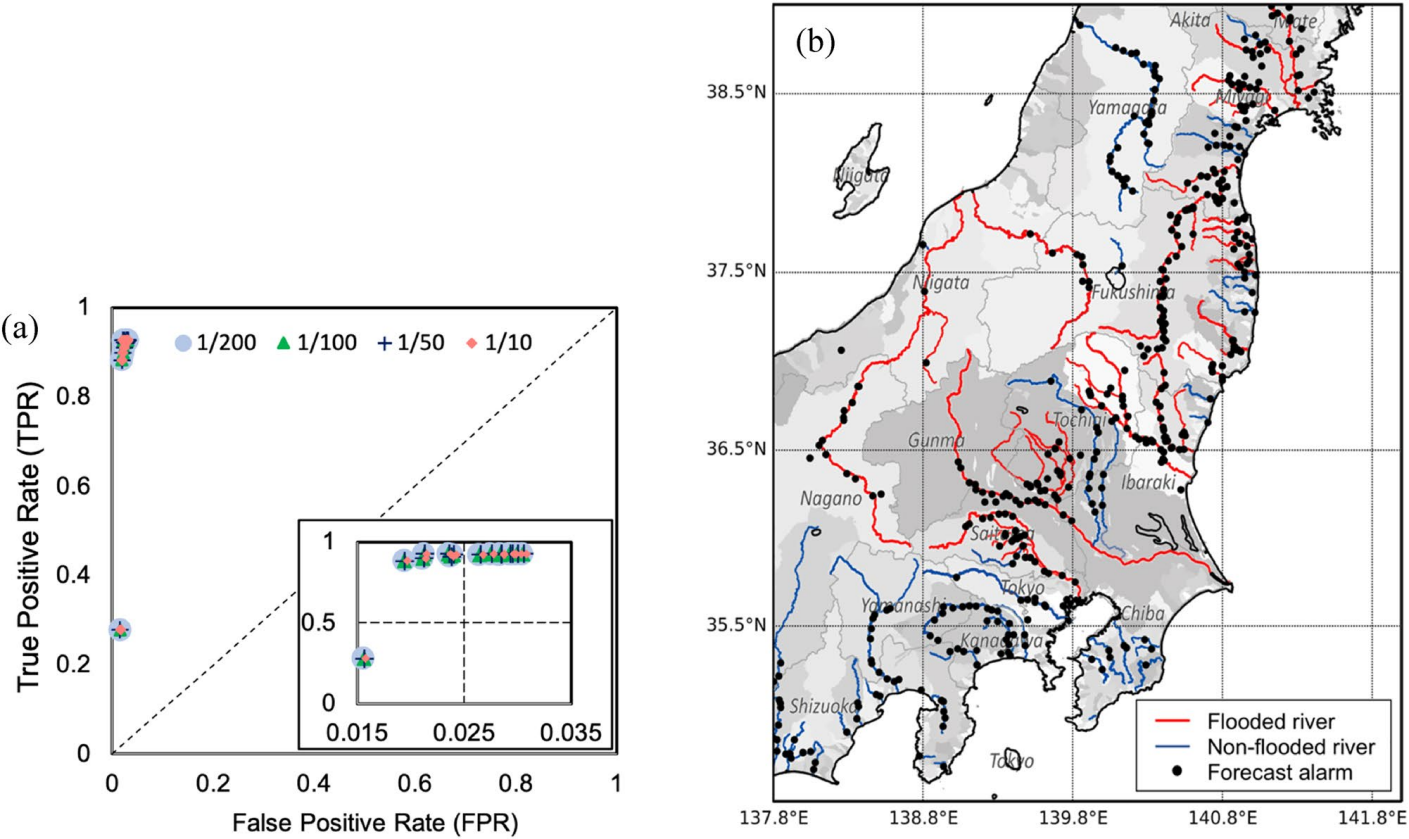
ROC curve for regions with precipitation in Japan (**a**) and the spatial distribution of forecast alarms and flooded major rivers due to Typhoon Hagibis (**b**). This figure was generated through Python2.7 (https://www.python.org/).

In addition to the successful alarms, we also analyzed false alarms, including false-positive (FP) locations. From the assessment of Typhoon Hagibis, we found that all 542 red pins (Fig. 1b) issued as indications of 1/200-year flooding achieved a hit ratio of 91.55% (130/142) and a precision of 24.0% (130/542). In addition, there were four FNWR locations (2.30%) where the system failed to forecast floods (Fig. 2). We plotted a relative operating characteristic (ROC) curve for an overview of forecast precision (Fig. 7a). In this study, the hit ratio indicates the probability of successfully forecasting flooded grids among all regions with precipitation. Consistent with the alarm setting in this flood forecasting system, we plotted the ROC curve by referring to discharge probability index (DPI) thresholds of 200, 100, 50, and 10 years every 3 h between 00:00 (JST) on October 11 to 12:00 on October 12, 2019. As shown in Fig. 7a, the FP rates were all distributed within a value of 0.03 for all dots for all thresholds, which indicates that the forecasting ability (when approaching the left corner of the plot) is good. The small FP rate indicates that FPs made up only a small proportion of true-negative (TN) locations at all time steps considered by this forecasting system. The TN locations were grids with precipitation but without alarms or observed flooding. Therefore, the results imply that the system did not provide false alarms for most non-flooded grids. The FP rate for 1/200-year floods was better than that for other values, indicating that the 1/200-year threshold was suitable for the case of Typhoon Hagibis, which produced a large amount of precipitation.

To check the false alarms, we evaluated them in two aspects. The first aspect was to check false alarms of *exact location but inexact time*, which is the most direct way to assess forecasting accuracy. To do so, we assessed forecast alarms issued for exact flooding locations (Figs. 4, 5). However, it is undeniable that insufficient data can cause assessment deviations. Furthermore, flood risk tends to increase naturally along lengthy parts of a channel, so a number of alarms covering a large area are forecast by our system. Nevertheless, when a dike break occurs at one location, it significantly decreases the chance of flooding at other areas. In such a case, with the *exact location but inexact time* aspect, most of the alarms are counted as false alarms and there is a single good alarm. Therefore, we also considered a second aspect of false alarms of *inexact location but exact time*, which has great significance for flood forecasting over a large area. Forecast alarms indicate high flood risk from rivers or catchments. To assess false alarms related to *inexact location but exact time*, we overlaid forecast alarms onto the relevant catchments, which is helpful for determining the spatial distribution of information. As shown in Fig. 7b, the plotted main streams of flooded rivers (red) and non-flooded rivers (blue) during Typhoon Hagibis were densely surrounded by forecast alarms. Most of these forecast alarms indicate risk areas for flooding near these 21 major rivers. Four major rivers actually had no flooding (blue), which was considered an overestimation in terms of a warning forecast. However, flooding was still possible around these four major rivers, because of a lack of observations or actual reports. In addition to being along major rivers, some alarms appeared near the outlets of streams or downstream from catchments (Fig. 7b). This study applied a 0.05-degree resolution, which is relatively coarse for the small channels that may coexist within one modeling grid. By comparing our spatial distribution with the flood locations shown in Fig. 3, it is obvious that some locations, such as those of eastern Fukushima, southern Miyagi, southern Chiba, and southwestern Shizuoka, shown as black dots in Fig. 7b, were actually not flooded. These alarms are reasonable selections for considering FP alarms of *exact location but inexact time* and *inexact location but exact time*. These FP locations may be attributable to one of the following three causes: the meteorological forcing data, hydrological model, or statistical analysis. In terms of meteorological forcing, higher precision^32^ and spatial resolution for each forecasted data point are required.

One possible way to improve flood forecasting accuracy is to adopt an ensemble prediction approach^16^, which should be analyzed in future work. Moreover, there is still opportunity for improvement in the resolution of the hydrological models used in forecasting systems. In this study, the results provided by CaMa-Flood have resolution of 0.05 degrees, which is the same as the resolution of EFAS and finer than that of other forecasting systems^6^. However, this resolution is relatively coarse for the many small channels that may coexist within one modeling grid. A finer resolution would produce a more reliable representation of hydrological states; however, it is subject to the resolution of the meteorological forcing data. In addition to the uncertainty due to forcing data and model resolution, some of the false alarms in this forecast might have been generated by underestimated 200-year return periods. Studies on the return period have demonstrated that analyzing homogeneous return periods may result in bias^33^. Although we used a 200-year return period as a threshold, the actual designed flood level exhibited particular variability. A survey and arrangement of designed flood level data are required in a future study.

Finally, validation is a great obstacle in flood forecasting because a shortage of observation data is a common difficulty. In particular, disaster monitoring systems are greatly challenged when disasters occur. Observation shortages may lead to underestimation of the accuracy of modeling and cause deviations in the validity of forecasted results. How to extend in situ observations remains a problem that can only be answered by taking cost into account. There is still potential for using satellite observations to enhance the quantitative information analyzed in hydrological studies.

In this paper, we present a flood forecasting system that is more useful in forecasting extreme flood events, such as the events of Typhoon Hagibis, compared to conventional forecasting based on gauged water levels. Despite system deficiencies, including limited modeling spatial resolution and forecasting precision, such long lead-time flood forecasting is urgently needed for early warning in Japan. At present, the JMA is issuing flood forecast alarms no earlier than 3 h ahead of time, which may result in difficulties in evacuation at night or for people who find it inconvenient to evacuate in such a short amount of time. A flood forecasting system with more than 30 h of lead time is helpful in many ways. Particularly, given the increasing tendency of extreme precipitation events worldwide^34–38^, an accurate flood forecasting technique is urgently needed in Japan and the rest of the world.

## Methods

### System description

This flood forecasting system was developed by Ishitsuka^18,19^. Its performance is shown in Supplementary Fig. 1. The modeling framework includes a land surface model, the MATSIRO^29^, and a global river routing model, CaMa-Flood^30^. The river water depth from CaMa-Flood was compared based on its statistical distribution across various return period values. The forecasting system makes hydrographic predictions for all rivers in Japan, which are integrated into a model mesh with 0.05-degree resolution.

MATSIRO is a physically based land surface model analyzing an environment consisting of a single-layer canopy, three layers of snow (at maximum), and six layers of soil. It simulates vertical movement of water and energy at the global scale. In Japan, it covers the area from 24° to 46° N latitude and 123° to 148° E longitude with a horizontal resolution of approximately 5 km (0.05 degrees)^29^. The input atmospheric forcing data include precipitation, temperature, surface pressure, wind speed, and radiation^20^. The output runoff from MATSIRO is used to run CaMa-Flood, the river routing model. CaMa-Flood was originally developed as a global hydrodynamic model that solves the local inertial Eq.^39^. It calculates the river discharge of a one-dimensional river channel with a rectangular riverbed and trapezoid floodplain storage. The river network, routing direction, and river parameters were calculated from the Multi-Error-Removed Improved-Terrain (MERIT) DEM and hydrography (MERIT Hydro)^21,22^ datasets with approximately 5-km (0.05-degree) horizontal resolution in Japan^30^. CaMa-Flood calculates river water depth ($D_{r}$) from the total water stored ($S_{r}$) at each grid point, as shown in Eq. 1:

1 $$D_{r} = \frac{{S_{r} }}{WL}$$

where *W* is the channel width, and *L* is the channel length. Each grid point has a river channel reservoir and a floodplain reservoir, which make up the unit catchment of the river channel.

### Forcing data preparation

In this study, MSM-GPV data^40,41^ provided by the JMA were used as meteorological forcing data. The MSM-GPV dataset includes 39-h forecast data around Japan, with a horizontal grid of 5 km and 50 vertical layers, which are released every 3 h (00, 03, .., 21 UTC). MSM-GPV data have been widely applied in meteorological and hydrological research in Japan on precipitation^42–44^ and typhoons^45^, wind^42,46^, energy^44–50^, and others^51–54^. In this study, we applied humidity, cloud cover, precipitation, surface air pressure, downward shortwave radiation, downward longwave radiation, wind speed/direction, and air temperature from the MSM-GPV dataset as meteorological forcing data. To minimize bias caused by precipitation, initial boundary data were estimated using radar data (Fig. 1) provided by the JMA at the same resolution. This method has been tested by Yoshimura et al.^20^.

### Data collection for alarm locations and DBTs

To assess the validity of the alarms forecasted by the model, we simply compared the alarm times and locations with the corresponding DBTs and locations of flood areas, which were obtained from the MLIT (http://xml.kishou.go.jp/xmlpull.html), JMA disaster prevention information in XML format (http://agora.ex.nii.ac.jp/cps/weather/river/), and NHK (https://www.nhk.or.jp/). The DBTs from MLIT and JMA were our primary data sources, as they provided the most reliable and rigorous information, including spatiotemporal details. The secondary source was the media (NHK), which had quickly broadcasted news of severe inundation, including the general location and timing. To prepare a systematic list of flood information, flood locations were mostly obtained from data from MLIT but supplemented by data from JMA and NHK.

### Statistical analysis

Significant systematic biases inevitably exist between naïve simulations and reality^26^ because of errors in the forcing data and inherent uncertainty in the models^55^. Instead of improving only the accuracy of forecasting, an accessible and practical method to mitigate the problem is to combine modeling results and statistical analysis because the results of hydrological modeling inevitably contain uncertainties^56–58^. Simulated results from a land surface model can reproduce hydrological processes to some extent. However, the output can be used more effectively when combined with statistical analysis. Many statistical distributions, such as the generalized extreme value distribution^59,60^, Gumbel distribution^23–27^, log-normal distribution^61,62^, and log Pearson type-III distribution^63^, have been tested in flood studies. Based on the characteristics of historical flood distributions, the Gumbel distribution^28^ is widely accepted as representing extreme flood events well because of its better fit in extreme value analysis^26^. Application of the Gumbel distribution was consistent with the estimation carried out by Yoshimura et al.^26^. First, the following equation was applied to estimate the probability distribution of the annual maximum discharge for each grid:

2 $$F_{\left( D \right)} = \exp \left[ { - \lambda \left( {1 - G_{\left( D \right)} } \right)} \right] = \exp \left( { - {\text{exp}}\left( { - \frac{D - \mu }{\beta }} \right)} \right)$$

where $F$ is the cumulative distribution function (CDF) of annual maximum values, *D* is the discharge, $G$ is the CDF of the values that exceed a specific threshold value, $\lambda$ is a constant representing annual occurrence frequency, and $\beta$ and $\mu$ are the scale and location parameters of the Gumbel distribution, respectively.

Second, the scale and location parameters of the Gumbel distribution were estimated as follows:

3 $$\hat{\beta } = \frac{1}{M}\mathop \sum \limits_{i = 1}^{M} \left( {D_{i} - D_{M} } \right), \hat{\mu } = D_{M} + \hat{\beta }\ln \lambda , \lambda = M/N$$

where $D$_*i*_ indicates the *i*th maximum and $M$ and $N$ are the numbers of samples and years, respectively, which give $\lambda$, a constant representing annual occurrence frequency. DPI (${\Pi }$) for all the daily maximum discharges was calculated as follows:

4 $${\Pi } = \left( {1 - F_{\left( D \right)} } \right)^{ - 1} = \left( {1 - {\text{exp}}\left( { - {\text{exp}}\left( { - \frac{D - \mu }{\beta }} \right)} \right)} \right)^{ - 1}$$

The unit for DPI is years, meaning that the probability of exceeding discharge $D$ in a year is 1/${\Pi }$ and the expected occurrence is once in ${\Pi }$ years if the discharge occurs at an annual maximum value.

In this study, 10 years of flood events were collected, and the Gumbel distribution was analyzed for each grid. The river water depth estimated by CaMa-Flood was compared with the 1/200-year flood water depth. For river water depths exceeding the 1/200-year threshold, alarms appeared in the forecasting system interface (Fig. 1b).

### ROC curve

The ROC curve is an effective way of assessing forecast ability in terms of hit rate and false warning rate. The ROC curve plots the proportion of occurrences that have been forecasted successfully (TP rate, y-axis) versus the proportion of false alarms (FP rate, x-axis) with reference to different thresholds^64–67^. In this study, the hit ratio (TP rate) indicates the probability of successfully forecasting grids (TP) among both TP and FN grids (Eq. 5). The false alarm rate refers to FP detection among both FP and TN grids (Eq. 6). The precision is indicated by the positive predictive value (PPV), defined in Eq. (7).

5 $$TPR = \frac{TP}{{TP + FN}}$$

6 $$FPR = \frac{FP}{{FP + TN}}$$

7 $$PPV = \frac{TP}{{TP + FP}}$$

Both the TP and FN rates were estimated from observation data. The values of the total grids including all negative and positive locations were selected from the grids with precipitation. According to the ROC plot, if the curve approaches the top-left corner, the forecasting system has a greater ability to forecast floods. Conversely, if the ROC curve lies close to the diagonal, the forecasting ability is considered weak^68^.

## Supplementary Information

Supplementary Information.

## Data Availability

The datasets generated during and/or analyzed during the current study are available in the “Zenodo” repository, (10.5281/zenodo.4604483).
