# Supplementary material for: Applicability of a nationwide flood forecasting system for Typhoon Hagibis 2019
Source: Sci Rep. 2021 May 13;11:10213. doi: 10.1038/s41598-021-89522-8 (PMC8119424; doi:10.1038/s41598-021-89522-8)
Supplement: Supplementary file 1 — Supplementary Information. [file 41598_2021_89522_MOESM1_ESM.docx]

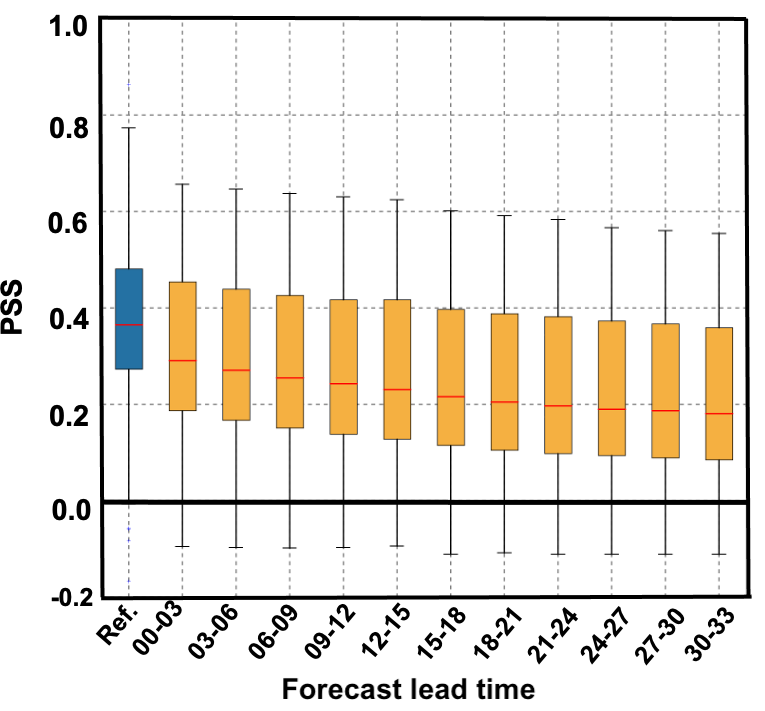


Supplementary Figure 1. The Pierce’s skill score (PSS) of the flood forecasting system based on the evaluation of 849 stations for forecasting performance from 2007 to 2017. Positive values indicate high levels of predictability for forecasting high water levels ^1,2^. More than 90% out of the 849 stations have a positive PPS with forecasts having a 33-h lead time. More than 50% of all stations possess a PSS larger than 0.25 with having a 12-h lead time.

PSS = $\frac{\mathbf{TP}}{TP+FN}-\frac{FP}{\mathrm{FP} +\mathrm{TN}}$

(S1)

# **References**

1. Addor, N., Jaun, S., Fundel, F. & Zappa, M. An operational hydrological ensemble prediction system for the city of Zurich (Switzerland): Skill, case studies and scenarios. Hydrol. Earth Syst. Sci. 15, 2327–2347 (2011).

2. Alfieri, L. et al. GloFAS-global ensemble streamflow forecasting and flood early warning. Hydrol. Earth Syst. Sci. 17, 1161–1175 (2013).
